# Watery Discharges of Pregnant Women

**Published:** 1886-02

**Authors:** Charles Warrington Earle

**Affiliations:** Professor of Obstetrics, College Physicians and Surgeons


THE WATERY-DISCHARGES OF PREGNANT WOMEN. *By* CHARLES WARRINGTON EARLE, M. D., *Professor of Obstetrics, College Physicians and Surgeons.*

[Read before the Chicago Medical Society.]

Mrs. F. K. consulted me for a profuse watery-discharge which had taken place several times during her pregnancy, commencing at the third month. She was the mother of three children, and had always been free from any marked pelvic disease. The first discharge was clear and watery, and she estimates the quantity at about two (2) quarts. This came away in a gush, most of it being discharged at once, although there was a slight loss for some days thereafter. At first it was thin and clear, then slightly thicker, with the color of weak coffee. These discharges seemed to occur every two or three weeks, and were frequently attended with considerable pain. There was a decided diminution in the size of her abdomen after each discharge.

On October 30th I found her in great pain, and an examination demonstrated that the fœtus was very low in the pelvis and apparently not surrounded with any liquor amnii. The os uteri was neither soft nor dilated. She was ordered anodynes and to remain in bed. On the 7th of November I again saw her, and found she had been having more or less pains since my previous visit. There was no dilatation. Anodyne and rest were prescribed. Two days after, however, she was delivered, her gestation having been continued about 200 days. The child lived about one hour. She made a good recovery and resumed her place in her family in the course of two weeks.

Mrs. M., aged 27; in her 9th pregnancy. At the end of five months she commenced to have a flow of fluid which continued until the end of the seventh month, when she gave birth to twins, one living and the other dead. There was no escape of liquor amnii at her confinement. The same lady in her eleventh pregnancy commenced to lose fluid at the end of the seventh month, which continued until the completion of the full term, when she gave birth to a healthy child. She had what her attendants called a dry-labor.

Mrs. D. W. R., aged 31, the mother of nine children, has been pregnant since the 1st of July, 1885. On November 20th she said to a friend who was at her bedside that she was flowing, and asked to be supplied with a napkin. A sheet was folded and placed under the patient, which was thoroughly saturated with fluid; the discharge being equal probably to at least two pints. She had severe pains which simulated those of labor, lasting a few hours. On December 15th she had a similar discharge. The future of this case is yet to be decided.

*Frequency.*—These cases evidently take place with more frequency than we have, up to this time, supposed; but the older obstetric authors have noticed peculiarities of this kind, and given very fair descriptions of the complication.

Smellie says, page 179, Vol. ii, "Dribbling of fluid may go on for weeks, but a sudden gush is invariably followed by parturition; the longest interval between a sudden gush and labor being seven days." In this he is certainly mistaken, as the history of many recorded cases and some of mine will demonstrate.

Denman, 1815, says: "Instances have been recorded in which the waters of the ovum are said to have been voided as early as the sixth month of pregnancy without prejudice either to the child or the mother. The truth of these reports seems to be doubtful, because where the membranes are intentionally broken, the action of the uterus never fails to come on. A few cases of this kind, somewhat similar, have occurred to me: A discharge of colorless fluid takes place, daily, from the vagina for several months preceding labour, which is due to the rupture of some lymphatic. Such labours are usually premature and the fœtus small."

The same authority also cites a case where, after the delivery of the placenta, several pints of lymph were discharged.

Burns, 1822, page 238, says that discharges of watery-fluid from the vagina are not infrequent, and generally depend upon the secretion of glands about the cervix; the rupture of lymphatics, or from fluid collected between the chorion and amnion, or water from blighted ovum in the case of twins.

Dr. Pentland relates a case where coughing produced a discharge, the water being discharged at the fourth month; but labor only occurred at full term.

Merriman, in his work entitled "Difficult Parturition," 1826, relates a case of a lady—six months pregnant—from whom a profuse, watery-discharge occurred. She summoned a physician, who assured her that if pains came on she would soon be delivered. She continued, however, to the end of pregnancy, having a profuse discharge each day. At full term she was delivered, her attending physician rupturing a bag of water, which appeared in no way different from usual cases. No opening was discoverable in either the placenta or the membranes, and he concluded that the discharge must have come from the outside of the membranes.

Chailly, edited by Bedford, 1844, gives a rather full account of hydrorrhœa, the description not being different from those I have already related. He says, however, that these discharges are more frequent than are generally supposed, but makes the erroneous statement that in nearly all these cases pregnancy is carried along to its full term.

Nearly all modern authors devote a short section to the consideration of this subject, giving different names, as their ideas of its origin and pathology are different.

Three separate pathological conditions seem to be, in many cases, confounded, and I see no way by which a differentiation can be made.

1st. A discharge of the liquor amnii.

2d. Discharges from increased glandular action.

3d. A possible collection of fluid between or outside of the membranes, and its irregular evacuation.

In my teachings I have been in the habit of speaking of hydrorrhœa, but never, up to a few months ago, had I seen a marked case. A study of this case with others collected from my own experience, and the perusal of the article written by Dr. Thomas C. Smith, of Washington, D.C., which appeared in the *Obstetric Journal* in May, has caused me to go over the subject carefully and to present what I can obtain from the authorities in regard to these peculiar discharges.

Great numbers of cases have been recorded, but no one, up to this time, has demonstrated conclusively the source of the flow.

The etiology of these discharges has been the subject of very different opinions by different obstetric authors. Chailly says that authors have attempted to show that these discharges are due to the accumulation of fluid between chorion and amnion; to rupture of lymphatic vessels; to transudation through amniotic membranes; to rupture of the membranes at some remote point from the orifice of the uterus, and finally, to dropsy of the womb.

Lusk says the pathological processes involved in the disease are vascularity, hyperæmia and hypertrophy of the interstitial connective-tissue, and of the glandular elements of the decidua.

Barnes, in the System of Obstetric Medicine and Surgery, 1885, says in regard to these discharges and without entering into a critical discussion of the several theories, that it seems to be well established that there are five sources from which this fluid may come :

1st. A discharge from the cervical canal.

2d. The decidual origin.

3d. Transudation through the amniotic membranes.

4th. Hydatidiform degeneration of the ovum.

5th. Cauliflower excrescences.

*Symptoms.*—There are no symptoms which give any indication that these discharges are about to take place, and after they have occurred once there is nothing which will give us intimation that they are about to recur. The pain is sometimes great, and at other times none will be experienced.

In a few cases, when the liquor amnii is drained off, there is a very perceptible diminution in the size of the abdomen, and the fœtus will be found low in the pelvis, almost as if it were wedged into the cavity. The color and the consistency of the fluid will be different in different cases.

The *Differential Diagnosis* must rest between the following similar discharges:

I. From the discharge from hypertrophied cervical glands.

II. Fluid collecting between chorion and amnion, occurring only once.

III. Escape of fluid from amniotic cavity.

I. The fluid escaping from the hypertrophied glands must be small in quantity, and we would expect that it would continue for a considerable length of time. There would be no diminution in the amount of liquor amnii and the child would be found floating in the usual amount of fluid.

II. If the fluid collected between any of the membranes, and adhesive-inflammation surrounded it followed, a considerable amount of fluid might collect, and the discharges would be considerable at once, and might or might not be repeated. In such a case there would be no evidence of escape of true amniotic fluid, although there might be a lessened size of the abdomen.

III. Where the liquor amnii escapes there would be a greater tendency to uterine contractions; a more perceptible diminution in the size of uterine tumor, and a microscopical or chemical examination would certainly reveal some evidence of urine, as we know this exists in variable quantities in the liquor amnii.

Transudation through the amniotic membrane, although recently noticed by Barnes, has been mentioned by older authors, would give rise to the discharge of a very small amount of fluid.

This could hardly be differentiated from a slight discharge, taking place from the cervical glands. Fluids discharged from hydatidiform degeneration of the chorion or from cauliflower excrescence, would be so associated with the diseases which cause them, that the diagnosis would not be difficult.

*Prognosis.*—As far as my observation goes the life of the woman is not jeopardized, but she suffers from the constant discharge and becomes anæmic. The pain is sometimes severe, as I have before remarked, and the patient is full of gloomy forebodings and anxious in regard to the final result.

The fœtus is usually born prematurely, and, in many cases, only lives a short time.

The treatment must necessarily be very simple—rest and anodynes being about all that can be suggested.

